# Celiac Disease Autoimmunity in Patients with Autoimmune Diabetes and Thyroid Disease among Chinese Population

**DOI:** 10.1371/journal.pone.0157510

**Published:** 2016-07-18

**Authors:** Zhiyuan Zhao, Jing Zou, Lingling Zhao, Yan Cheng, Hanqing Cai, Mo Li, Edwin Liu, Liping Yu, Yu Liu

**Affiliations:** 1 Department of Endocrinology, The Second Hospital of Jilin University, Changchun, Jilin, China; 2 Barbara Davis Center for Childhood Diabetes, University of Colorado Denver, Aurora, Colorado, United States of America; Renal Division, Peking University First Hospital, CHINA

## Abstract

The prevalence of celiac disease autoimmunity or tissue transglutaminase autoantibodies (TGA) amongst patients with type 1 diabetes (T1D) and autoimmune thyroid disease (AITD) in the Chinese population remains unknown. This study examined the rate of celiac disease autoimmunity amongst patients with T1D and AITD in the Chinese population. The study included 178 patients with type 1 diabetes and 119 with AITD where 36 had both T1D and AITD, classified as autoimmune polyglandular syndrome type 3 variant (APS3v). The study also included 145 patients with type 2 diabetes (T2D), 97 patients with non-autoimmune thyroid disease (NAITD), and 102 healthy controls. Serum islet autoantibodies, thyroid autoantibodies and TGA were measured by radioimmunoassay. TGA positivity was found in 22% of patients with either type 1 diabetes or AITD, much higher than that in patients with T2D (3.4%; p< 0.0001) or NAITD (3.1%; *P* < 0.0001) or healthy controls (1%; p<0.0001). The patients with APS3v having both T1D and AITD were 36% positive for TGA, significantly higher than patients with T1D alone (p = 0.040) or with AITD alone (p = 0.017). T1D and AITD were found to have a 20% and 30% frequency of overlap respectively at diagnosis. In conclusion, TGA positivity was high in the Chinese population having existing T1D and/or AITD, and even higher when both diseases were present. Routine TGA screening in patients with T1D or AITD will be important to early identify celiac disease autoimmunity for better clinical care of patients.

## Introduction

Autoimmune type 1 diabetes (T1D) and autoimmune thyroid disease (AITD) are common organ-specific autoimmune endocrine diseases. Their pathogenesis involves the specific T lymphocyte-mediated autoimmune destruction in a specific target organ and the corresponding specific autoantibodies can be detected in the blood. T1D and AITD are important components of autoimmune polyglandular syndrome (APS). APS is an autoimmune disease involving dysfunction of more than one endocrine gland [1]. Autoimmune polyglandular syndrome type 3 variant (APS3v) is a subtype of APS characterized by the simultaneous or successive development of specifically AITD and T1D [2].

Celiac disease (CD) is defined as a chronic small intestinal immune-mediated enteropathy precipitated by exposure to dietary gluten in genetically predisposed individuals. Its classic presentation includes diarrhea, abdominal pain, and abdominal distension caused by chronic intestinal malabsorption, although some individuals may have extra-intestinal features as the primary presentation. Furthermore, patients identified through screening as having celiac disease may not have clinically apparent symptoms even though they may have or be at risk of celiac-related complications. The disease-specific transglutaminase autoantibodies (TGA) can be detected in the serum as an early marker of CD autoimmunity. The incidence of CD is very high at 1:100 to 1:300 in North America, Scandinavia, and Australia [3, 4]. The incidence of CD is even higher in patients with T1D, ranging from 5–10% in the Caucasian population [5, 6], and in patients with AITD, is 10 times greater than that in the general population [7–10]. The prevalence of CD in the Chinese population has never been studied, and has traditionally thought to be rare. However, a recent report [11] found that although the frequencies of HLA DQ2 and DQ8 haplotypes were lower than that in the United States, they were not insignificant (3.4% and 2.1% respectively), meaning that a subpopulation in China could be at higher risk of CD. In this report, we investigated the prevalence of TGA in a specific Chinese population that should be considered to be at a higher risk–those with T1D and/or AITD [12, 13] and evaluated the frequency of TGA positivity indicating CD autoimmunity.

## Materials and Methods

### Study subjects

In total, 178 patients with T1D with disease duration of less than 12 months were enrolled in this study. Diabetes was diagnosed in accordance with 1999 World Health Organization diagnostic criteria for diabetes at Jilin University Hospital in China from 2010 to 2013. The islet autoantibodies to glutamic acid decarboxylase-65(GAD65), insulinoma-associated protein-2(IA-2), and zinc transporter 8(ZnT8) were used to confirm the diagnosis of T1D. We also studied 119 patients with AITD with disease duration of less than 3 months. The diagnostic criterion for AITD was having positive thyroid autoantibodies including thyroid-stimulating hormone receptor autoantibodies (TRAb), thyroid peroxides autoantibodies (TPOAb), and/or thyroglobulin autoantibodies, with either abnormal or normal thyroid function, including chronic autoimmune thyroiditis or Hashimoto's thyroiditis, painless thyroiditis, atrophic thyroiditis or primary hypothyroidism and Graves' disease. Of 297 patients in total, 36 with both T1D and AITD were classified as APS3v. In the study, we also included 145 patients with type 2 diabetes (T2D) with disease duration of less than 12 months, as well as 97 patients with non-autoimmune thyroid disease (NAITD) with disease duration of less than 3 months. Finally, 102 age-matched healthy subjects without a family history of diabetes or an autoimmune disease history were included as controls. Patients with heart, brain, liver, and kidney disease and other organic diseases were excluded. Informed consent was obtained from all participants studied. All procedures performed in the study were approved by the Institutional Ethics Committee of the Second Hospital of Jilin University in accordance with the 1964 Declaration of Helsinki declaration and its later amendments or comparable ethical standards.

### Autoantibody assays

Serum was collected from whole blood and frozen at -80°C for use in all assays. Detection of TPOAb was conducted using a specific commercial kit (Weifang 3V Biological Engineering Group Co., Ltd., Weifang, China) with positive cutoff value of 12 U/L and detection of TRAb was also performed with a specific commercial kit (Tianjin Xiehe Medical Technology Group Ltd., Tianjin, China) with positive cutoff value of 40 IU/ml. Detection of thyroglobulin antibodies was done using a specific commercial kit (Siemens Healthcare Diagnostics Inc., Massachusetts, USA) with positive cutoff value of 60 U/ml. These commercial kits are commonly used for clinical diagnosis in hospitals and clinics in China. The radioligand binding assays for measuring islet autoantibodies to zinc transporter 8 (ZnT8A), insulinoma-associated protein-2 (IA-2A), and glutamic acid decarboxylase (GADA), and transglutaminase autoantibodies (TGA) were adopted from Barbara Davis Center for Childhood Diabetes University of Colorado as previously described and reported as an index [12, 14, 15]. A ZnT8A index of ≥0.033, GADA index of ≥0.089, IA-2A index of ≥0.058, and TGA index of ≥0.025 were considered positive and the cutoff index of these autoantibodies were established as the 99th percentile of 102 control samples. The inter-assay coefficient variations were 8.1%, 8.6%, 5.5% and 9.8% for ZnT8A, IA-2A, GADA and TGA, respectively.

### Statistical Analysis

The data were analyzed using SPSS 21.0 software (IBM Corp., Armonk, NY, USA) and expressed as mean ± standard deviation or median (range). The rate and constituent ratio between groups were compared using the χ^2^ test or Fisher’s exact test (when the theoretical frequency ranged from 1 to <5, the continuity-corrected χ^2^ test was used; when the theoretical frequency was <1 or the total case number was <40, Fisher’s exact test was used). Correlation analysis between groups was performed using Spearman’s rank correlation coefficient. A *P* value of <0.05 was considered statistically significant.

## Results

**Fig 1:**
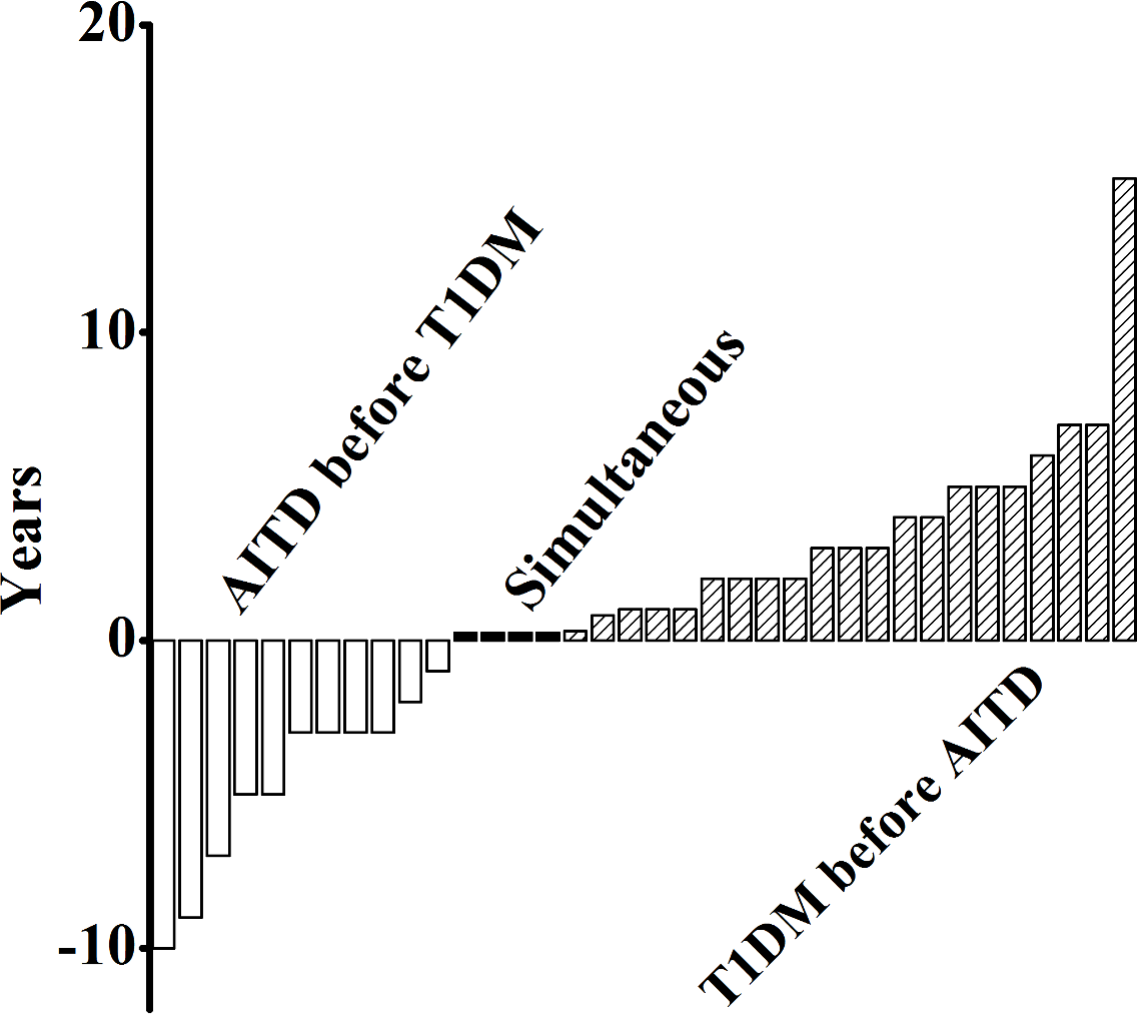
The interval from the onset of T1D to the onset of AITD or vise versus in APS3v patients. The open, closed, and shaded bars respectively represent the individual patients who developed AITD before, simultaneous or after T1D diagnosis.

Amongst all individuals with T1D, 20.2% (36/178) had associated AITD, and amongst all individuals with AITD, 30.25% (36/119) had associated T1D. These individuals with both T1D and AITD were classified as APS3v. Basic characteristics of the study participants were shown in Table 1. Among the patients with APS3v, 58% (21/36) of patients developed T1D before AITD and 30.6% (11/36) of patients were diagnosed with AITD before T1D. T1D and AITD were diagnosed simultaneously in four patients. The longest onset interval was 15 years (Fig 1).

**Table 1:** Clinical data of subjects.

	T1D	T2D	AITD	NAITD	APS3v	Control
	(n = 142)	(n = 145)	(n = 83)	(n = 97)	(n = 36)	(n = 102)
**Gender** (male/female)	66/76	84/61	13/70	21/76	12/24	44/58
**Age** (year)	27.2±15.2	42.3±15.6*	35.4±15.4*	35.6±13.7*	48.0±16.2*	26.3±3.5
**BMI** (kg/m^2^)	21.4±5.6^#^	25.3±4.1*	23.3±3.2*	21.9±4.4^#^	23.3±5.5*	19.7±1.7
**FBG** (mmol/l)	13.6±7.1^*^	12.2±4.3*	4.8±0.5	4.8±0.7	10.3±4.8*	4.8±0.4

Compared with control group

^#^ *P*<0.01

^*^ *P*<0.001.

**Fig 2:**
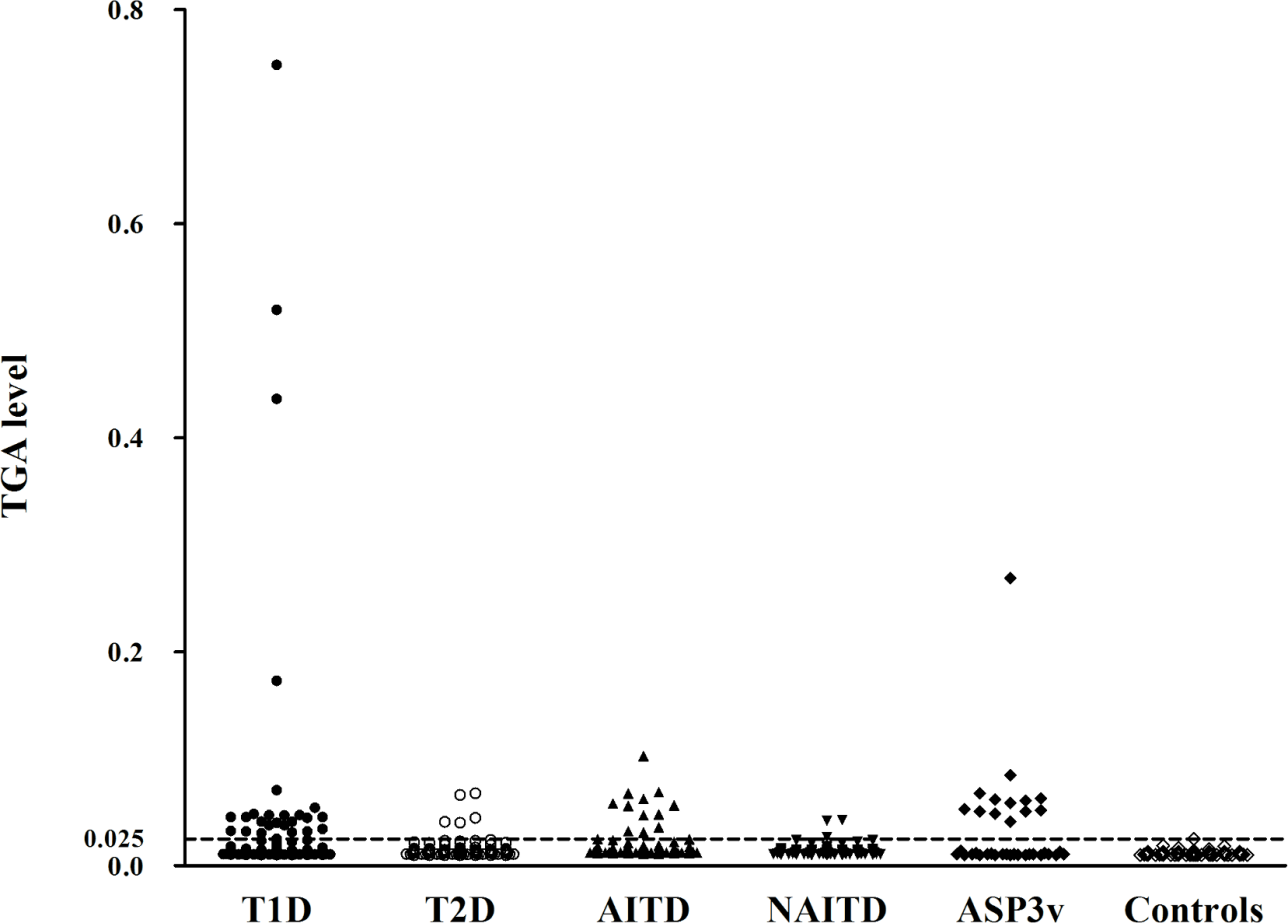
TGA levels in patients with T1D, T2D, AITD, NAITD and Controls. Data are expressed as antibody index of individual subjects from T1D, T2D, AITD, NAITD and Controls. The dotted line represents the cutoff value of TGA.

**Fig 3:**
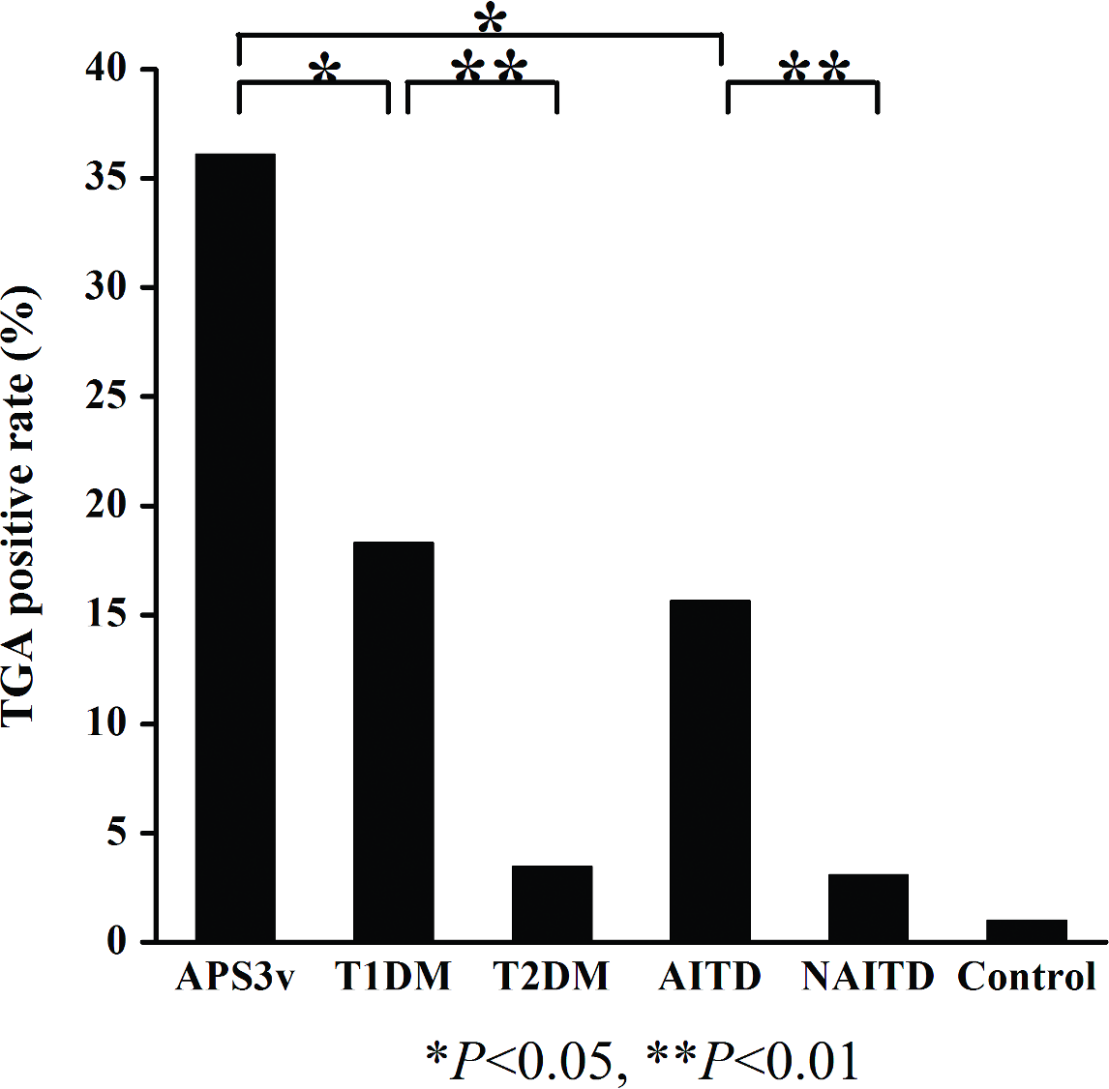
TGA positivity in patients with APS3v, T1D, T2D, AITD, NAITD and Controls. *P<0.05; **, *P*<0.01.

The levels of TGA in all patient groups and healthy controls were illustrated in Fig 2. With the cut-off set on 99^th^ percentile of 102 control samples, the prevalence of TGA in patients with T1D was 22% (39/178), significantly higher than the healthy control group (1/102; p<0.0001). The prevalence of TGA in patients with AITD was also 22% (26/119), significantly higher than healthy controls (p<0.0001). These two cohorts were compared to their non-autoimmune counterparts–those with T2D and NAITD. The prevalence of TGA in patients with T2D (5/145) or NAITD (3/97) were not different from the healthy control group while TGA positivity in patients with T1D or AITD was significantly higher than in patients with T2D (p<0.0001) and in patients with NAITD patients (p<0.0001), respectively. Interestingly, the prevalence of TGA in patients with APS3v having both T1D and AITD was up to 36% (13/36), significantly higher than that in patients with T1D alone (p = 0.040) or with AITD alone (p = 0.017) (Fig 3).

**Fig 4:**
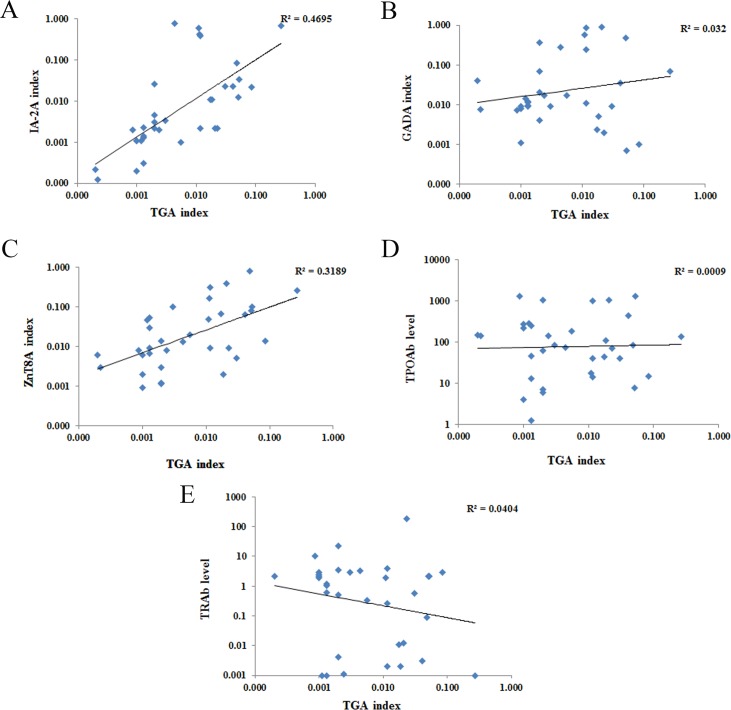
Correlation between TGA index and anti-islet autoantibody index among patients with APS3v. A-C represents correlation between TGA and IA-2A, GADA, ZnT8A, respectively; D-E shows the correlation between TGA and TPOAb, TRAb, respectively.

The analysis of correlation between the levels of TGA and islet autoantibodies or thyroid autoantibodies was shown in the Fig 4. Interestingly, the TGA levels were shown to significantly correlate with the levels of IA-2A (R^2^ = 0.187, p<0.05) and weakly correlate with ZnT8A levels, but not significantly (R^2^ = 0.107, p = 0.058). There are no level correlations of TGA with GADA or with any thyroid autoantibodies including TRAb and TPOAb. Also no correlations were seen in levels between islet autoantibodies and thyroid autoantibodies (data not shown).

All patients in this study received a CD symptom questionnaire and we found a significant connection in patients with TGA positive to clinical symptoms of CD such as abdominal pain, diarrhea, nausea, vomiting, and weight loss. Table 2 showed the incidence of CD related clinical symptoms in patients with/ without TGA positivity. The prevalences of CD symptoms were significantly higher in patients with TGA positive compared to those with TGA negative, either in patients with autoimmune T1D and/or AITD (63.5%[33/52] versus 20.1% [42/209]; p<0.001) or in patients with non-autoimmune T2D or NAITD (75%[6/8]versus 12.8%[30/234]; *p*<0.001). However, among patients with/without TGA positive, the prevalence of CD symptoms in patients with autoimmune T1D or AITD showed no difference from those with non-autoimmune T2D or NAITD.

**Table 2:** Incidence of CD symptom among patients with/without TGA positive.

	Autoimmune diseases	Non-autoimmune diseases (N = 242)	*P* value
	(N = 261)		
TGA+	63.5%(33/52)	75.0%(6/8)	0.881
TGA-	20.1%(42/209)	12.8%(30/234)	0.079
*P* value	<0.001	<0.001	

## Discussion

To our knowledge, screening and epidemiological studies of CD autoimmunity in China have not been performed, although it has been reported to occur in the population [11]. In this study, we have identified a high prevalence of TGA positivity in patients with T1D and/or AITD among Chinese population, 22% in patients with either T1D or AITD and 36% in those with both T1D and AITD.

TGA positivity was strongly associated with autoimmune T1D and AITD, but not with non-autoimmune T2D or NAITD. The TGA positivity in T2D and NAITD was near the background, similar to the healthy control group, suggesting that the CD autoimmunity is not associated with dysregulation of glucose metabolism in diabetes or dysfunctioning of thyroid gland with thyroid disease, but is associated with existing autoimmunity. It is clearly shown that different organ-specific autoimmune diseases are greatly overlapped. In the present study, over 20% of patients with T1D were found having AITD and over 30% of patients with AITD had T1D. The results demonstrate that the age of diagnosis of APS3v is much higher than the age of onset of T1D and AITD due to the onset interval of these two different diseases. The interval which can be defined as the duration of APS3v ranges from months to years according the onset of T1D and AITD. Horie et al. [16] reported that the longest onset interval was >30 years in patients with concurrent Graves’ disease and T1D.

CD and T1D share the high risk HLA Class II of DR3-DQ2 and DR4-DQ8, and non-HLA genetic susceptibility in Caucasian population and their clinical phenotypes overlap in up to 10% of the patients [12]. AITD in the Caucasian population also has DR3-DQ2 as its highest risk HLA haplotype [13].

This study is limited by the fact that the TGA-positive patients were not able to have a final diagnosis of CD by small intestinal endoscopy and biopsy even though symptoms were more common in those with TGA positivity as shown in Table 2. In addition, TGA positivity does not necessarily indicate CD, but represent a significant step towards the CD autoimmune process that may lead to CD in this high-risk screened population. The small sample capacity might probably cause the notably elevated TGA positivity in patients with T1D and AITD. Further clinical follow-up data of TGA-positive patients will be important to confirm persistence of the autoantibodies and possible future development of CD. In addition, genotyping of patients in the Chinese population will also be helpful to identify both HLA and non-HLA genes contributing to the presence of organ-specific autoimmunity and shared between these three different organ-specific autoimmune diseases.

In conclusion, CD autoimmunity of TGA-positivity was found high in patients with autoimmune T1D and/or AITD among Chinese population and these different organ-specific autoimmunities were greatly overlapped each other. The regular screening in patients with one autoimmune disease for autoantibodies of other autoimmune diseases will be important for the clinical care of patients and may provide some insights into the pathogenesis of these complex diseases.

## Supporting Information

S1 File Autoantibodies levels of subjects included in this study. (XLSX)

## References

[1] Eisenbarth GS, Gottlieb PA. Autoimmune polyendocrine syndromes. N Engl J Med. 2004;350(20):2068–79. 10.1056/NEJMra030158 . 15141045

[2] Huber A, Menconi F, Corathers S, Jacobson EM, Tomer Y. Joint genetic susceptibility to type 1 diabetes and autoimmune thyroiditis: from epidemiology to mechanisms. Endocr Rev. 2008;29(6):697–725. 10.1210/er.2008-0015 18776148 PMC2583387

[3] Ciclitira PJ, Johnson MW, Dewar DH, Ellis HJ. The pathogenesis of coeliac disease. Mol Aspects Med. 2005;26(6):421–58. 10.1016/j.mam.2005.05.001 . 16125764

[4] Fasano A, Berti I, Gerarduzzi T, Not T, Colletti RB, Drago S, et al Prevalence of celiac disease in at-risk and not-at-risk groups in the United States: a large multicenter study. Arch Intern Med. 2003;163(3):286–92. . 12578508 10.1001/archinte.163.3.286

[5] Dube C, Rostom A, Sy R, Cranney A, Saloojee N, Garritty C, et al The prevalence of celiac disease in average-risk and at-risk Western European populations: a systematic review. Gastroenterology. 2005;128(4 Suppl 1):S57–67. . 15825128 10.1053/j.gastro.2005.02.014

[6] Agardh D, Nilsson A, Tuomi T, Lindberg B, Carlsson AK, Lernmark A, et al Prediction of silent celiac disease at diagnosis of childhood type 1 diabetes by tissue transglutaminase autoantibodies and HLA. Pediatr Diabetes. 2001;2(2):58–65. 10.1034/j.1399-5448.2001.002002058.x . 15016199

[7] Ch'ng CL, Jones MK, Kingham JG. Celiac disease and autoimmune thyroid disease. Clin Med Res. 2007;5(3):184–92. 10.3121/cmr.2007.738 18056028 PMC2111403

[8] Norstrom F, Sandstrom O, Lindholm L, Ivarsson A. A gluten-free diet effectively reduces symptoms and health care consumption in a Swedish celiac disease population. BMC Gastroenterol. 2012;12:125 10.1186/1471-230X-12-125 22984893 PMC3482575

[9] Viljamaa M, Kaukinen K, Huhtala H, Kyronpalo S, Rasmussen M, Collin P. Coeliac disease, autoimmune diseases and gluten exposure. Scand J Gastroenterol. 2005;40(4):437–43. . 16028438 10.1080/00365520510012181

[10] Guariso G, Conte S, Presotto F, Basso D, Brotto F, Visona Dalla Pozza L, et al Clinical, subclinical and potential autoimmune diseases in an Italian population of children with coeliac disease. Aliment Pharmacol Ther. 2007;26(10):1409–17. 10.1111/j.1365-2036.2007.03526.x . 17892522

[11] Yuan J, Gao J, Li X, Liu F, Wijmenga C, Chen H, et al The tip of the "celiac iceberg" in China: a systematic review and meta-analysis. PLoS One. 2013;8(12):e81151 10.1371/journal.pone.0081151 24324669 PMC3852028

[12] Bao F, Yu L, Babu S, Wang T, Hoffenberg EJ, Rewers M, et al One third of HLA DQ2 homozygous patients with type 1 diabetes express celiac disease-associated transglutaminase autoantibodies. J Autoimmun. 1999;13(1):143–8. 10.1006/jaut.1999.0303 . 10441179

[13] Tomer Y. Mechanisms of autoimmune thyroid diseases: from genetics to epigenetics. Annu Rev Pathol. 2014;9:147–56. 10.1146/annurev-pathol-012513-104713 24460189 PMC4128637

[14] Wenzlau JM, Moua O, Sarkar SA, Yu L, Rewers M, Eisenbarth GS, et al SlC30A8 is a major target of humoral autoimmunity in type 1 diabetes and a predictive marker in prediabetes. Ann N Y Acad Sci. 2008;1150:256–9. 10.1196/annals.1447.029 . 19120307

[15] Yu L, Cuthbertson DD, Maclaren N, Jackson R, Palmer JP, Orban T, et al Expression of GAD65 and islet cell antibody (ICA512) autoantibodies among cytoplasmic ICA+ relatives is associated with eligibility for the Diabetes Prevention Trial-Type 1. Diabetes. 2001;50(8):1735–40. . 11473032 10.2337/diabetes.50.8.1735

[16] Horie I, Kawasaki E, Ando T, Kuwahara H, Abiru N, Usa T, et al Clinical and genetic characteristics of autoimmune polyglandular syndrome type 3 variant in the Japanese population. J Clin Endocrinol Metab. 2012;97(6):E1043–50. 10.1210/jc.2011-3109 . 22466347

